# PPARδ regulates satellite cell proliferation and skeletal muscle regeneration

**DOI:** 10.1186/2044-5040-1-33

**Published:** 2011-11-01

**Authors:** Alison R Angione, Chunhui Jiang, Dongning Pan, Yong-Xu Wang, Shihuan Kuang

**Affiliations:** 1 Department of Animal Sciences, Purdue University, 901 West State Street, West Lafayette, IN 47907, USA; 2 Program in Gene Function and Expression and Program in Molecular Medicine, University of Massachusetts Medical School, 364 Plantation Street, Worcester, MA 01605, USA; 3 Center for Cancer Research, Purdue University, 201 S. University Street, West Lafayette, IN 47907, USA

**Keywords:** Cre/LoxP, skeletal muscle, stem cell, proliferation, differentiation, self-renewal

## Abstract

Peroxisome proliferator-activated receptors (PPARs) are a class of nuclear receptors that play important roles in development and energy metabolism. Whereas PPARδ has been shown to regulate mitochondrial biosynthesis and slow-muscle fiber types, its function in skeletal muscle progenitors (satellite cells) is unknown. Since constitutive mutation of *Pparδ *leads to embryonic lethality, we sought to address this question by conditional knockout (cKO) of *Pparδ *using *Myf5-Cre/Pparδ^flox/flox ^*alleles to ablate PPARδ in myogenic progenitor cells. Although *Pparδ*-cKO mice were born normally and initially displayed no difference in body weight, muscle size or muscle composition, they later developed metabolic syndrome, which manifested as increased body weight and reduced response to glucose challenge at age nine months. *Pparδ*-cKO mice had 40% fewer satellite cells than their wild-type littermates, and these satellite cells exhibited reduced growth kinetics and proliferation *in vitro*. Furthermore, regeneration of *Pparδ*-cKO muscles was impaired after cardiotoxin-induced injury. Gene expression analysis showed reduced expression of the *Forkhead box class O transcription factor 1 *(*FoxO1*) gene in *Pparδ*-cKO muscles under both quiescent and regenerating conditions, suggesting that PPARδ acts through FoxO1 in regulating muscle progenitor cells. These results support a function of PPARδ in regulating skeletal muscle metabolism and insulin sensitivity, and they establish a novel role of PPARδ in muscle progenitor cells and postnatal muscle regeneration.

## Background

Skeletal muscle is the most abundant tissue in mammals, making up 45% to 55% of total body mass in humans, and plays important roles in body movement and metabolic regulation. Muscle is made up of different fiber types which have different metabolic requirements that affect the whole body energy homeostasis of the animal [1]. Type 1 fibers are classified as slow fibers and use oxidative metabolism as a fuel source, making them highly fatigue-resistant. Conversely, type 2 fibers are classified as fast fibers, use mainly glycolytic metabolism and are less resistant to fatigue. Type 2 fibers are further broken down into three subtypes, known as types 2a, 2x and 2b, that express corresponding myosin heavy chain (MyHC) isoforms and have decreasing resistance to fatigue. Notably, skeletal muscles are plastic, and fiber-type switching occurs in response to changes in activity and other physiological signaling pathways [2-4]. In addition, skeletal muscle mass is always in a state of hypertrophy or wasting, based on relative use or disuse, respectively [5,6]. Skeletal muscle has superior capacity to regenerate itself upon injury [7].

Skeletal muscle plasticity is mainly maintained by a subset of cells known as satellite cells [8,9]. These cells, located beneath the basal lamina of the muscle fiber, are normally maintained in a quiescent state. Satellite cells become activated when the muscle becomes damaged through injury or normal activity. Once activated the cells will reenter the cell cycle and undergo a few rounds of division, then differentiate and fuse with existing muscle fibers to rebuild the damaged area. Satellite cells in the quiescent state express paired-box transcription factor 7 (Pax7) [10]. After activation the cells will express Pax7 and myogenic differentiation antigen 1 (MyoD) concurrently while the cells undergo a few rounds of division (proliferation). These proliferating cells eventually withdraw from the cell cycle and either return to quiescence (self-renewal) through downregulation of MyoD or differentiate through downregulation of Pax7 and upregulation of myogenin. Thus expression of Pax7 and MyoD distinguishes the status of a cell, whether it is self-renewing (Pax7^+^/MyoD^-^), proliferating (Pax7^+^/MyoD^+^) or differentiating (Pax7^-^/MyoD^+^), respectively [11-13]. Notably, researchers in several recent studies have demonstrated that the choice between self-renewal and differentiation of newly divided satellite cells is dynamically regulated [14-16]. Whereas most proliferating myoblasts divide symmetrically, a subpopulation of cells can divide asymmetrically to give rise to both self-renewal and differentiating progenies [14-16].

Myogenic factor 5 (Myf5) is also important for skeletal muscle development and satellite cell function. *Myf5 *transcripts can first be detected at E8 in the developing embryo and are important for specifying the cells of the muscle lineage [17]. *Myf5-Cre *lineage tracing labels a majority of satellite cells, which will have become committed to the myogenic lineage. However, 10% of satellite cells remain *Myf5*-negative and are thought to be a population of more primitive, uncommitted stem cells that can give rise to committed cells through asymmetric cell division [15]. *Myf5*-null satellite cells are defective in transient proliferation prior to differentiation [18,19]. Intriguingly, investigators in recent studies have demonstrated that brown adipocytes, but not white adipocytes, are derived from Myf5 lineage progenitors [20]. Therefore, *Myf5-Cre*-mediated conditional knockout can be used to knock out floxed target genes in committed myogenic progenitor cells and their descendants (mature skeletal muscles) as well as in brown adipocytes.

Peroxisome proliferator-activated receptors (PPARs) are members of the nuclear estrogen receptor superfamily and have been shown to be important for the proper metabolism of fatty acids [21]. There are three PPAR isoforms (α, δ and γ), and each plays a specific role in metabolism [22-24]. The PPARs are expressed in a wide range of adult tissues, but each has its own tissue-specific expression patterns. PPARα is highly expressed in the liver and heart, PPARδ (also referred to as PPARβ, PPARβ/δ or NR1C2) is highly expressed in the intestine and liver, and PPARγ is highly expressed in both brown and white adipose tissues [21-23,25,26]. The three PPAR isoforms are ligand-activated receptors that are activated by fatty acids, fatty acid derivatives and a variety of synthetic compounds [21]. The ligand-binding domains of PPARs vary slightly, resulting in specific affinity for fatty acids and synthetic compounds [27]. For example, GW501516 is one synthetic compound that has been shown to specifically activate PPARδ with 60- to 1, 000-fold selectivity over the other isoforms, depending on cell types and animal species [28,29]. These receptors must form heterodimers with the retinoid X receptor (RXR) before they can bind to specific recognition sequences, called PPAR response elements (PPREs), which are located in the promoter and intron regions of a wide variety of target genes [21,30]. The PPARs activate or repress transcription through the recruitment of coactivators and corepressors.

PPARδ has been shown to be important for the proper function of skeletal muscle in gain- and loss-of-function studies. Synthetic compound-mediated activation or overexpression of the *Pparδ *gene in mice causes an increase in the oxidative capacity of the muscle resulting from an increase in the number of type 1 oxidative fibers and a decrease in the number of type 2 glycolytic fibers [31,32]. These increases in oxidative capacity have been shown to contribute to an organism's overall exercise endurance. Along with increases in oxidative capacity, transgenic mice also show a decrease in overall body fat content and individual adipocyte size. Mice with constitutively active *Pparδ *can maintain a normal body weight even when challenged with a high-fat diet, whereas their wild-type littermates become obese when fed the same diet. By contrast, skeletal muscle-specific knockout of *Pparδ *seems to cause a decrease in oxidative capacity and makes mice prone to obesity and metabolic disorders [33]. This loss of oxidative capacity could be due in part to reduced expression of PGC1α, which is known to play a role in type 1 fiber formation and maintenance. These results suggest that PPARδ plays a role in preventing obesity and the development of metabolic disorders.

Until now all the studies on PPARδ in skeletal muscle have focused on mature muscle fibers. It remains to be determined whether PPARδ plays a role in myogenic satellite cells and postnatal muscle regeneration. PPARδ has been shown to regulate the proliferation and/or maturation of several cell types, including mouse embryonic stem cells, oligodendrocytes, keratinocytes, endothelial progenitors and cancer cells [34-38]. However, whether PPARδ positively or negatively regulates proliferation is highly cell type-specific, and the evidence presented in the literature has sometimes been contradictory [36]. In the current study, we used a Cre/LoxP-based conditional mutation approach to remove *Pparδ *from the satellite cells to examine its function in muscle progenitor cells. We show herein that PPARδ is an important regulator of satellite cell proliferation *in vitro *and of muscle regeneration *in vivo*.

## Materials and methods

### Animals

All experimental procedures involving the use of mice were carried out in accordance with Purdue University's Animal Care and Use Committee. C57BL/6J mice containing LoxP sites flanking exon 4, which encodes the N-terminal zinc finger of the DNA-binding domain of the *Pparδ *gene (*Pparδ*^f/f^), had been generated previously [39]. These mice were crossed with Myf5-Cre to generate Myf5-Cre/*Pparδ*^f/f ^offspring for ablation of *Pparδ *in *Myf5 *lineage cells (called "*Pparδ*-cKO" hereinafter) [40]. *Pparδ*^+/f ^or *Pparδ*^f/f ^littermates that did not inherit the Myf5-Cre allele were used as controls (called "wild type" hereinafter, as these mice express *Pparδ *normally). Genotyping was done by PCR to confirm the presence of Cre along with the presence of floxed and wild-type *Pparδ *alleles as described by The Jackson Laboratory under mouse stock numbers 007893 and 005897 (Bar Harbor, ME, USA).

### Cardiotoxin injection

Tibilais anterior (TA) muscles taken from six-week-old C57BL/6J mice were injured by injection of cardiotoxin (CTX) (C3987; Sigma-Aldrich, St Louis, MO, USA) to induce muscle regeneration. The animals were first anesthetized by intraperitoneal injection of 0.2 ml ketamine cocktail/20 g body weight. Ketamine cocktail contains 0.9 ml of ketamine (100 mg/ml), 0.1 ml of xylazine (100 mg/ml) and 9.0 ml of saline. The hind limbs were then shaved to expose the belly of the TA and wiped with 70% ethanol. Next we injected 50 μl of 10 μM CTX into the belly of the TA muscle. The mice were allowed to recover on a heating pad for about one hour. The mice were then harvested at days 5 and 14 after injection, and their TA muscles were removed for RNA extraction and histological examination after being fixed in 4% paraformaldehyde.

### Glucose challenge

Glucose tolerance tests were performed on two- and nine-month-old mice. All mice were fasted for three hours prior to the start of testing. Blood was collected from the tip of the tail and tested before (0 minutes) and 15, 30, 60 and 90 minutes after intraperitoneal injection of glucose (2 g/kg). Blood glucose levels were measured using an ACCU-CHEK Active blood glucose meter system (Roche Diagnostics, Indianapolis, IN, USA). Body weight data were collected prior to glucose tolerance testing.

### Cell culture

Primary myoblasts were harvested from the limb muscles of mice that were six weeks of age. Limb muscles were digested in a solution containing 1% collagenase B (Roche Diagnostics) and 2.4 U/ml Dispase II (neutral protease, grade II; Roche Applied Science) for 30 minutes in an incubator at 37°C with 5% CO_2 _and triturated every 15 minutes. The digestion was stopped after 30 minutes using 25 ml of DMEM containing 2% fetal bovine serum (FBS) and 10 mM 4-(2-hydroxyethyl)-1-piperazineethanesulfonic acid, then passed through a 100-μm filter. After filtration, the cells and muscle debris were pelleted at 250 × *g *for five minutes and the DMEM was removed. The cells and muscle debris were then plated on noncoated plates with Ham's complete medium containing 20% FBS, 4 ng/ml basic fibroblast growth factor and 1% penicillin/streptomycin (p/s) (10, 000 U penicillin/g/ml, 10 mg streptomycin/ml). The cells and muscle debris were maintained in an incubator at 37°C with 5% CO_2 _for three days, and 5 ml of Ham's medium were added each day. On the third day, all cells and muscle debris (with cells attached) were collected into a 15-ml conical tube and digested with 1 ml of 0.025% trypsin for five minutes at 37°C. Dissociated cells were resuspended in 10 ml of Ham's complete medium and passed through a 30-μm filter, then plated on a collagen-coated plate. Myoblasts were maintained in Ham's medium and passed through the filter several times before they became senescent and were discarded. To differentiate myoblasts, Ham's medium was switched to DMEM containing 5% horse serum with 1% p/s when cultures reach 80% confluence. For cell-growth analysis, primary myoblasts from both *Pparδ*-cKO and wild-type mice were counted, and 50, 000 cells were seeded into each well of a six-well plate. Cells were removed from the plate with trypsin and counted with a hemocytometer on days 3, 6 and 9 after the initial plating.

### Isolation and culture of single myofibers

Single myofiber-carrying satellite cells were isolated as previously described [41]. Fibers were harvested from the soleus (SOL) and extensor digitorum longus (EDL) muscles of mice that were six weeks of age. Whole muscles were removed from the hind limbs by careful handling of the tendons only. The SOL and EDL muscles were first placed in 5 ml of DMEM containing 0.2% collagenase I and then into the water bath at 37°C for 40 minutes (EDL muscle) or 80 minutes (SOL muscle). The fibers were then placed into a 6-cm plate with 5 ml of DMEM and separated from the tendons by careful manipulation using heat-polished Pasteur pipettes coated with horse serum to prevent sticking. After the fibers were separated, they were fixed immediately for staining or transferred onto a new plate containing 5 ml of DMEM with 20% FBS and 2% chick embryo extract, then placed in the incubator at 37°C with 5% CO_2 _for three days. At the end of the three days, the fibers were collected, fixed with 2% paraformaldehyde and prepared for staining.

### Immunocytochemistry

Primary myoblasts were grown in chamber slides for staining. The cells were fixed with 2% paraformaldehyde for ten minutes, then washed three times with PBS before the primary antibody was added. The primary antibodies used were Pax7 (Developmental Studies Hybridoma Bank, Department of Biology, University of Iowa, Iowa City, IA USA), MyoD (sc-20; Santa Cruz Biotechnology, Santa Cruz, CA, USA), and mAb clones HB287, HB277 and HB283 for MyHC1, MyHC2a and MyHC2b, respectively (American Type Culture Collection, Manassas, VA, USA). Cells were incubated with the primary antibody for one hour at room temperature on a shaker. After incubation with the primary antibody, the cells were washed three times with PBS before the secondary antibody was added. The secondary antibodies used were goat anti-mouse immunoglobulin G2b (IgG2b) conjugated with Alexa Fluor 647 dye, goat anti-mouse IgG1 conjugated with Alexa Fluor 568 dye, goat anti-mouse IgM conjugated with Alexa Fluor 488 dye (Molecular Probes/Life Technologies, Carlsbad, CA, USA) and daylight Alexa Fluor 488-conjugated goat anti-rabbit (Jackson ImmunoResearch Laboratories, Inc, West Grove, PA, USA). The cells were incubated with secondary antibody and Hoechst dye (a DNA dye) for 30 minutes. The cells were again washed three times with PBS before being mounted with Dako Fluorescence Mounting Medium (Glostrup, Denmark) and covered with a coverslip. Images were recorded on a Leica DMI6000 B inverted fluorescence microscope (Leica Microsystems, Mannheim, Germany).

### Histology

The muscles harvested from the hind limbs were embedded in optimal cutting temperature (O.C.T.) compound (Sakura Finetek USA Inc, Torrance, CA, USA) and quickly frozen in dry ice-cooled isopentane. Cryosections were cut to 10-μm thickness and placed on glass slides. For immunohistochemistry, slides were blocked with blocking buffer (5% horse serum, 2% BSA, 0.2% Triton X-100 and 0.1% sodium azide in PBS) for two hours prior to antibody staining. The slides were incubated with primary antibodies for one hour at room temperature, then washed three times with PBS before incubation with secondary antibody for 30 minutes. After three more washes with PBS, the slides were mounted with Dako Fluorescence Mounting Medium and covered with a coverslip.

### H & E and NADH staining

Sections (10-μm thickness) were first washed with PBS to remove excess O.C.T. compound, then incubated in hematoxylin for five minutes, washed and incubated in 1% eosin for 30 seconds. The sections were then dehydrated in increasing concentrations of ethanol and mounted with CytoSeal Mounting Medium (Electron Microscopy Sciences, Fort Washington, PA, USA), covered with a coverslip and allowed to dry overnight. For nicotinamide adenine dinucleotide, reduced (NADH) staining, sections were washed briefly in PBS to remove excess O.C.T. compound. The slides were then incubated in a solution containing 100 mg/ml NADH and 0.1 g/ml nitroblue tetrazolium (NBT) for 30 minutes at 37°C. The slides were then washed three times with deionized water. Unbound NBT was removed from the sections by washing the slides three times each with 30%, 60% and 90% acetone. The sections were then washed with deionized water and mounted.

### Gene expression

All gene expression data were gathered by real-time PCR. RNA was extracted from cell culture, whole-muscle and adipose tissue using the RNeasy RNA extraction kit (QIAGEN, Germantown, MD, USA). RNA was converted into cDNA using Moloney murine leukemia virus reverse transcriptase and random hexamer primers. The real-time PCR oligonucleotides are listed in Additional file 1, Table S1. Samples were then run in a LightCycler 480 System (Roche Applied Science) for 40 cycles. Fold changes were calculated using the 2^-ΔΔCt ^method.

### Western blot analysis

Expression of PPARδ protein in *Pparδ*-cKO and wild-type muscles was examined by Western blot analysis as previously described [32]. Briefly, equal amounts of nuclear proteins extracted under identical conditions were run on SDS-PAGE gels and transferred onto polyvinylidene difluoride membrane. PPARδ was detected with rabbit anti-PPARδ antibody from Santa Cruz Biotechnology (SC-7197).

### Statistical analysis

The number of mice used for each experiment is listed in the figure legend. Every experiment was performed in triplicate. For the glucose tolerance tests, statistical analysis was based on area under the curve (AUC) using the trapezoidal rule. All data are presented as means ± SEM. *P*-values were calculated using a two-tailed Student's *t*-test unless otherwise indicated. All values equal to 0.05 or less were considered significant and are denoted by asterisks in the figures.

## Results

### Characterization of the Pparδ-cKO model

To generate tissue-specific knockout of *Pparδ*, we used mice bearing a floxed exon 4 in the *Pparδ *gene [39]. The presence of Cre will excise exon 4, corresponding to the N-terminal zinc finger of the DNA binding domain of PPARδ, and lead to premature stop of translation and abolish the transcriptional activity of the resulting truncated PPARδ (Additional file 2, Figure S1A). The *Pparδ^f/f ^*mice were bred to mice expressing Cre recombinase under the control of the endogenous *Myf5 *promoter *Myf5-Cre *(007893; The Jackson Laboratory) to generate the *Pparδ*-cKO mice. Since *Myf5 *is expressed in the mesoderm during development, we expected that PPARδ would be selectively ablated in several mesodermal tissues that express *Myf5*, including skeletal muscle and brown fat (BAT) [20].

**Figure 1:**
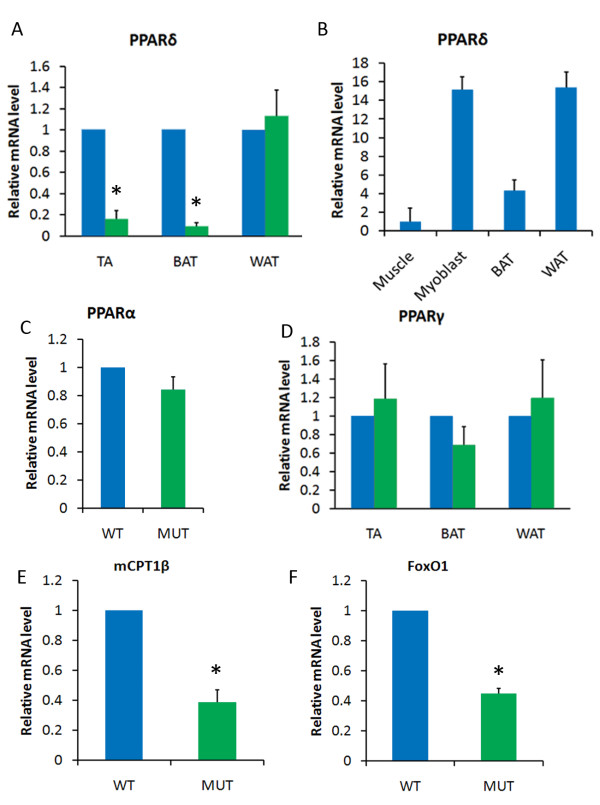
**Characterization of the PPARδ-cKO model**. Blue and green bars represent wild-type (WT) and peroxisome proliferator-activated receptor δ conditional knockout (*Pparδ*-cKO) (MUT) tissues, respectively. **(A) **Relative expression levels of *Pparδ *in tibialis anterior (TA) muscle, brown fat and white fat tissues (*N *= 8 pairs of wild-type and mutant mice) for each tissue type. **(B) **Quantitative RT-PCR showing the relative expression levels of *Pparδ *in whole-muscle tissue, proliferating myoblasts, white fat and brown fat in wild-type mice (*N *= 8 for each tissue type and *N *= 2 for myoblasts). **(C) **and **(D) **Expression levels of *Pparα *in TA muscle **(C) **and *Pparγ *in TA, brown fat and white fat tissues (*N *= 8 for each tissue type) **(D)**. **(E) **and **(F) **Expression levels of *carnitine palmitoyltransferase 1β *(*mCPT1β*) and *Forkhead box class O transcription factor 1 *(*FoxO1*) genes in TA muscles (*N *= 3).

To confirm the tissue-specific knockout, we collected RNA samples from adult skeletal muscle, BAT and white fat (WAT) and conducted quantitative RT-PCR (qPCR) analysis. The levels of *Pparδ *in the *Pparδ*-cKO mice compared to their wild-type littermates were reduced by 7-fold in TA muscle and by 11-fold in BAT, but they were not changed in WAT (Figure 1A). These results confirm the tissue-specific knockout and are consistent with the notion that skeletal muscle and BAT derive from the Myf5 lineage and WAT derives from a Myf5-independent lineage [20]. We further confirmed by Western blot analysis that PPARδ protein levels are reduced by 60% to 80% in the gastrocnemius muscles of *Pparδ*-cKO mice compared to those of wild-type mice (Additional file 2, Figure S1B). Interestingly, analysis of relative *Pparδ *expression in various wild-type tissues indicated that both proliferating myoblasts (derived from satellite cells) and WAT expressed much higher levels of *Pparδ *compared to whole muscle and BAT (Figure 1B). Specifically, *Pparδ *mRNA levels in primary myoblasts were about 15-fold greater than those in the TA muscles, suggesting a specific role of PPARδ in myoblast proliferation. In addition, we found that the levels of *Pparγ *and *Pparα *remained unchanged in *Pparδ*-cKO compared to wild-type tissues (Figures 1C and 1D), confirming that *Pparδ*-cKO did not elicit compensation by other PPAR isoforms. Therefore, any observed phenotypes are due to specific knockout of *Pparδ *in our mouse model.

To understand how PPARδ functions in muscle, we compared the expression of a number of known PPARδ target genes in wild-type and mutant TA muscles from adult mice. The expression of *carnitine palmitoyltransferase 1β *(*mCPT1β*), whose protein regulates the rate-limiting step for β-oxidation of long-chain fatty acids [21,24], and *Forkhead box class O transcription factor 1 *(*FoxO1*), a PPARδ target that regulates skeletal muscle metabolism and progenitor cell function [42-44], were significantly decreased in *Pparδ*-cKO muscle (Figures 1E and 1F). The expression of several other known target genes of PPARδ, including *Sirt1*, *UCP1 *and *PGC1α*, was not altered in the mutant muscles (Additional file 3, Figure S2). These results confirm that *Pparδ*-cKO affects candidate target gene expression and identify *mCPT1β *and *FoxO1 *as potential target genes regulated by PPARδ in resting skeletal muscles.

### Satellite cell and myoblast proliferation and differentiation

**Figure 2:**
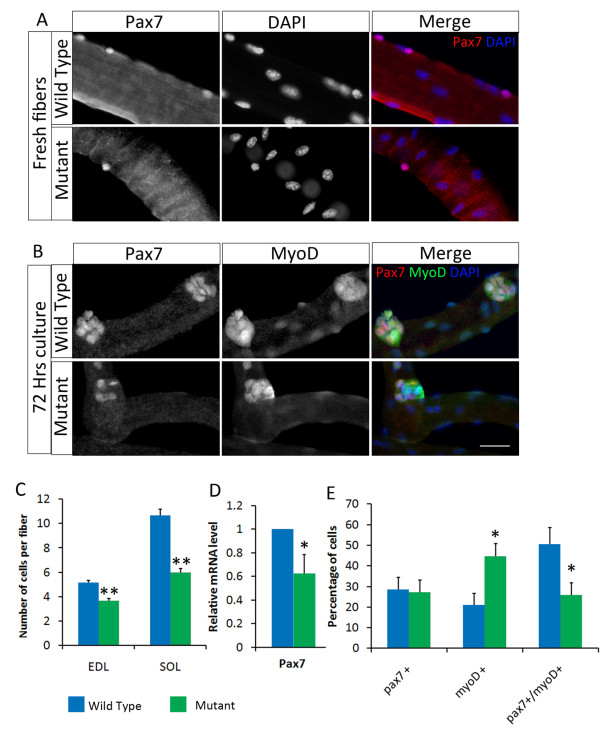
**Reduced satellite cell number and proliferation in *Pparδ*-cKO mice**. **(A) **Freshly isolated extensor digitorum longus (EDL) muscle fibers were stained with paired-box transcription factor 7 (Pax7) antibody to label satellite cells. Nuclei were counterstained with 4', 6-diamidino-2-phenylindole (DAPI). Scale bar = 25 μm for all panels in parts **(A) **and **(B)**. **(B) **Soleus (SOL) muscle fibers were cultured for 72 hours, and the proliferating satellite cells on the fibers were fixed and stained with Pax7 and myogenic differentiation antigen 1 (MyoD) antibodies. Nuclei were counterstained with DAPI. Satellite cell status is classified as self-renewed cells (Pax7^+^), proliferating cells (Pax7^+^/MyoD^+^) and differentiating cells (MyoD^+^). **(C) **Quantification of the number of Pax7^+ ^satellite cells per single fiber isolated from the EDL and SOL muscles (*N *= 360 fibers each from the SOL and EDL muscles). **P < 0.0001. **(D) **Relative expression of the *Pax7 *gene in tibialis anterior (TA) muscles by quantitative PCR (*N *= 3). **(E) **Quantification of the number of self-renewed, proliferating and differentiating cells on SOL fibers after culture for 72 hours (*N *= 20 fibers for each group).

Adult *Pparδ*-cKO mice have a reduced number of satellite cells, and *Pparδ*-null myoblasts exhibited reduced proliferation and increased differentiation kinetics. Because proliferating myoblasts expressed high levels of *Pparδ *compared to whole muscles (Figure 1B), we investigated the effect of *Pparδ *mutation on the satellite cells *in vivo *and in myoblasts in culture. Intact single fibers were isolated from the representative slow (SOL) and fast (EDL) muscles and stained with Pax7 antibody to label satellite cells (Figure 2A). At two to three months old, the mutant mice had, on average, a 40% reduction in satellite cell numbers in both EDL and SOL muscles (Figure 2C). Consistent with this observation, qPCR analysis indicated that satellite cell-specific *Pax7 *gene expression was reduced by about 40% in the mutant compared to the wild-type TA muscles (Figure 2D). Interestingly, normal satellite cell numbers were detected at three weeks of age, and a nearly 20% reduction was detected at age five weeks in the *Pparδ*-cKO compared to the wild-type EDL fibers (Additional file 4, Figure S3). Furthermore, the number of 4', 6-diamidino-2-phenylindole-positive nuclei per myofiber was reduced in the *Pparδ*-cKO mice at five weeks old but not at three weeks old (Additional file 4, Figure S3). The gradual reduction in satellite cells and differentiated myonuclei in the *Pparδ*-cKO muscle during postnatal growth suggests that PPARδ is important for satellite cell proliferation and maintenance.

Next we examined whether the satellite cells associated with the isolated fibers were able to proliferate and differentiate normally using a single-myofiber culture paradigm to mimic satellite cell activation *in vivo *[13,15]. We cultured isolated fibers for three days *in vitro *and fixed and stained clusters of myoblasts that had proliferated on single fibers with antibodies to Pax7 and MyoD (Figure 2B). Previous studies have established that Pax7^+^/MyoD^-^, Pax7^+^/MyoD^+ ^and Pax7^-^/MyoD^+ ^cells represent self-renewing, proliferating and differentiating progenies, respectively [11-13]. The relative percentage of cells in these three categories was examined. Compared to the wild type, mutant SOL fibers had half as many proliferating cells (Pax7^+^/MyoD^+^) and twice as many differentiating cells (MyoD^+^), whereas the proportion of self-renewing cells (Pax7^+^) remained the same between wild-type and mutant fibers (Figure 2E). Together, these results show that PPARδ is important for maintaining the proliferation of activated myoblasts and that loss of PPARδ leads to accelerated myogenic differentiation.

**Figure 3:**
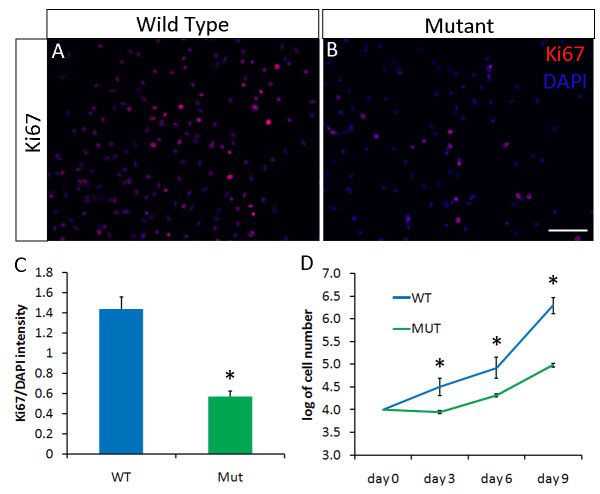
**Defective growth and proliferation of primary myoblast cells derived from *Pparδ*-cKO muscles**. **(A) **and **(B) **Representative images of Ki67 staining that specifically labeled proliferating primary myoblasts: Ki67 staining (red) and 4', 6-diamidino-2-phenylindole (DAPI) staining (blue). Scale bar = 50 μm. **(C) **Relative Ki67 signal intensity in wild-type and mutant myoblast cultures. The intensity of Ki67 and DAPI staining and the number of pixels were measured using Photoshop software (Adobe Systems Inc, San Jose, CA, USA). The ratio of Ki67 intensity values to DAPI intensity values was also quantified using Photoshop (*N *= 3). **(D) **Growth curve of wild-type and mutant myoblasts after nine days in culture (*N *= 3).

To further characterize the proliferative defects of *Pparδ*-null myoblasts, we examined the expression of Ki67, a cell proliferation marker, in cultured primary myoblasts from mutant mice and wild-type littermates (Figure 3A-B). Real-time PCR analysis confirmed a 60% reduction in *Pparδ *expression in newly established cultures of *Pparδ*-cKO compared to wild-type myoblasts (Additional file 5, Figure S4A). The percentage of Ki67^+ ^cells and the Ki67 immunofluorescence intensity of the *Pparδ*-mutant myoblasts were threefold less than those of wild-type plates (Figures 3C). Conversely, a PPARδ agonist, GW501516, significantly increased wild-type myoblast proliferation (Additional file 5, Figure S4B). We also plotted the growth curve of wild-type and mutant myoblasts at days 3, 6 and 9 after they were plated in culture. Myoblasts from *Pparδ*-cKO muscle showed a reduced growth rate compared to myoblasts from wild-type muscle (Figure 3D). These results provide compelling evidence that PPARδ positively regulates myoblast growth and proliferation.

### Muscle regeneration after injury is impaired in Pparδ-cKO animals

**Figure 4:**
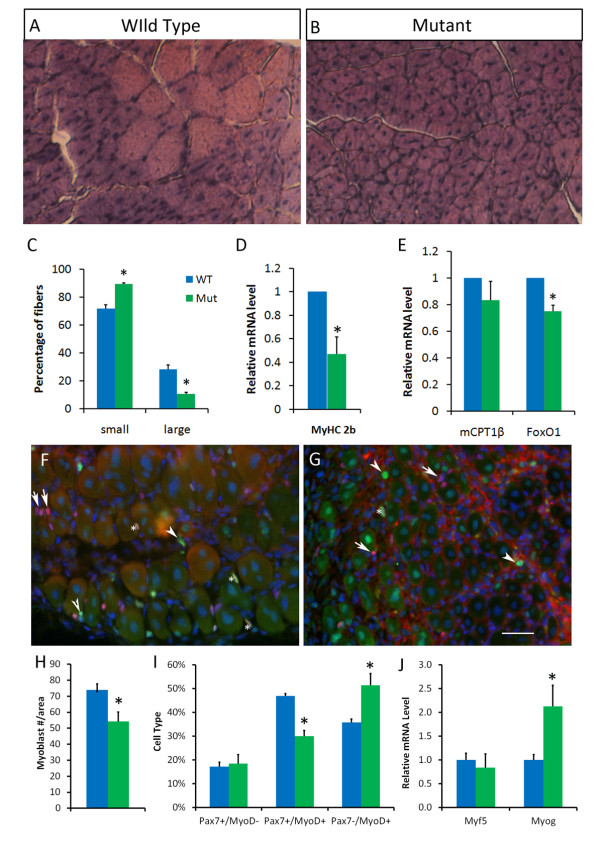
**Defective regeneration of skeletal muscles and gene expression patterns in *Pparδ*-cKO mice after injury with cardiotoxin**. **(A) **and **(B) **Tibialis anterior (TA) muscles from six-week-old wild-type and peroxisome proliferator-activated receptor δ conditional knockout (*Pparδ*-cKOmice were injected with cardiotoxin (CTX) to induce injury. The mice were allowed to recover for 14 days before their muscles were harvested and processed for staining. Small and large fibers (indicating regenerating and nonregenerating fibers, respectively) were counted. Scale bars = 50 μm for parts **(A) **and **(B) **and for parts **(F) **and **(G)**. **(C) **Quantification of regenerating and nonregenerating fibers after injury with CTX (*N *= 3 pairs of mice, with five sections taken from each mouse). **(D) **Relative mRNA expression level showing reduced myosin heavy chain isoform 2b (MyHC-2b) in mutant muscle after injury with CTX (*N *= 4). **(E) **Relative mRNA expression levels of *Pparδ *target genes in the TA muscle after injury with CTX (*N *= 4). **(F) **and **(G) **Cryosections of regenerating wild-type **(F) **and mutant **(G) **mice at day 5 after injury with CTX. The sections are labeled with paired-box transcription factor 7 (Pax7) (red), myogenic differentiation antigen 1 (MyoD) (green) and 4', 6-diamidino-2-phenylindole (DAPI) (blue). Arrows, arrowheads and asterisks indicate examples of Pax7^+^/MyoD^-^, Pax7^-^/MyoD^+ ^and Pax7^+^/MyoD^+ ^myoblasts, respectively. **(H) **The number of total myoblasts labeled by Pax7 and/or MyoD per unit area counted from 8 to 13 areas. **(I) **Percentage distribution of Pax7^+^/MyoD^-^, Pax7^+^/MyoD^+ ^and Pax7^-^/MyoD^+ ^myoblasts in regenerating wild-type and mutant TA muscles at day 5 after injury with CTX. **(J) **Relative mRNA expression levels of *Myf5 *and *Myogenin *in TA muscles at day 3 after injury with CTX (*N *= 3). *P < 0.05 by single-tailed Student's *t*-test.

To examine whether the reduced rates of proliferation in the *Pparδ*-cKO myoblasts result in defects in muscle regeneration *in vivo*, we injured the TA muscles of mutant and wild-type animals at six weeks of age. After 14 days, the mice were killed and the TA muscles were collected for gene expression and histological analysis. We examined the regenerating regions under a microscope, and the number of regenerating fibers (small, centrally located nucleus) and regenerated fibers (large, eccentrically located nucleus) were counted. Both wild-type and *Pparδ*-cKO TA muscles underwent regeneration, but they exhibited marked differences (Figures 4A and 4B). The mutant muscles had much larger areas of small-caliber regenerating fibers that covered almost the whole muscle sections, whereas the wild-type TA muscles had reduced regenerating areas that were mostly confined to the center of the section (Figures 4A and 4B). The number of small regenerating fibers was 30% more, and the number of large regenerating fibers was 20% fewer, in *Pparδ*-cKO muscles compared to wild-type muscles (Figure 4C). In addition, the regenerating *Pparδ*-cKO TA muscles expressed reduced levels of *MyHC 2b *mRNA, the most abundant MyHC isoform in the TA muscle (Figure 4D), which further confirms the regenerative defects. The *Pparδ*-cKO muscles were eventually able to regenerate after 30 days, suggesting that *Pparδ*-cKO mainly causes a delay in muscle regeneration.

Because *Pparδ*-cKO myoblasts are defective in proliferation *in vitro*, we sought to determine whether such defects exist *in vivo *during muscle regeneration. We analyzed Pax7 and MyoD expression (Figures 4F and 4G) at days 3 to 5 post-CTX treatment, at which stage myoblast proliferation peaks. Consistent with our *in vitro *results, we observed a reduction of proliferating Pax7^+^/MyoD^+ ^myoblasts and an increase in differentiated Pax7^-^/MyoD^+ ^myocytes in the *Pparδ*-cKO muscle, together with reduced total myoblast number per unit area and decreased size of newly regenerated fibers (Figures 4F to 4I). In addition, we confirmed by qPCR that *Myogenin *gene expression was upregulated in regenerating *Pparδ*-cKO muscles at this stage (Figure 4J). By contrast, *Myf5 *was expressed at similar levels in the mutant and wild-type muscles. These results indicate that the observed regenerative defects of *Pparδ*-cKO may be due to reduced proliferation and increased differentiation kinetics of activated myoblasts.

We also examined the expression of *mCPT1β *and *FoxO1 *genes during muscle regeneration at day 5 after CTX treatment, when damaged muscles are not yet regenerated and satellite cell proliferation peaks [8]. Interestingly, expression of *mCPT1β *was unchanged but *FoxO1 *expression was reduced in the *Pparδ*-cKO muscles compared to wild-type muscles during muscle regeneration (Figure 4E), suggesting that PPARδ may activate *FoxO1 *in proliferating myoblasts and that *mCPT1β *is a PPARδ target only in mature muscle fibers. Together with our earlier observation that *Pparδ *is expressed at much higher levels in proliferating myoblasts compared to mature muscles (Figure 1B), these results suggest a critical role of PPARδ in satellite cell function *in vivo*.

### Pparδ-cKO does not alter myosin heavy chain isoforms

**Figure 5:**
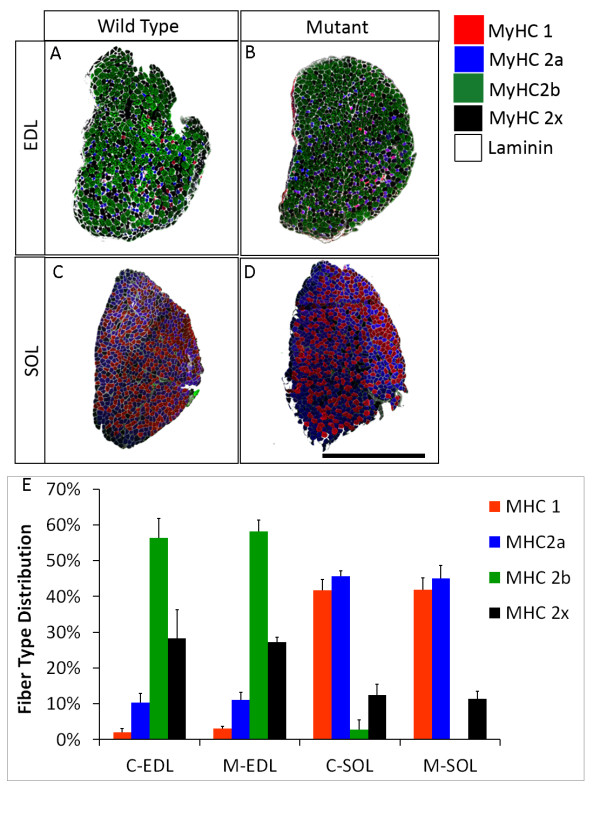
**Normal fiber-type distributions in extensor digitorum longus and soleus muscles of six-week-old *Pparδ*-cKO mice**. **(A) **through **(D) **Immunostained sections showing the distribution of fiber types in the extensor digitorum longus (EDL) and soleus (SOL) ("C-" denotes wild-type littermate control and "M-" denotes *Pparδ*-cKO): myosin heavy chain isoform 1 (MyHC-1) (red), MyHC-2a (blue), MyHC-2b (green), MyHC-2x (black) and laminin (white). The basal lamina surrounding each fiber is shown. Scale bar = 1 mm. **(E) **Percentage distribution of each fiber type in the EDL and SOL muscles (*N *= 4 pairs of littermates.

Because *Pparδ *overexpression increases oxidative muscle fiber types [32], we sought to determine whether *Pparδ*-cKO causes any changes in fiber-type distribution. We labeled MyHC protein isoforms with a panel of mAbs (Figures 5A to 5D). We chose the EDL and SOL muscles to represent fast and slow muscle types, respectively. To overcome potential bias associated regionalized distribution of fiber types within a muscle [45], we enumerated all muscle fibers in the entire muscle (Figures 5A to 5D). No difference in the percentage of each fiber type was found between mutant and wild-type animals in either the EDL or SOL muscle at six weeks old (Figure 5E). To further determine whether there are changes in muscle oxidative metabolism without changing MyHC isoform expression, we carried out NADH-tetrazolium reductase (NADH-TR) staining of the SOL and EDL muscles. NADH-TR staining marks ATPase activity that is correlated to oxidative capacity (type 1 fibers are highly oxidative and darkly stained, type 2a fibers are intermediate fibers and lightly stained and type 2b fibers are glycolytic and unstained) (Additional file 6, Figures S5A to S5D). In agreement with our MyHC isoform staining experiments, there were no differences in NADH activity between the *Pparδ*-cKO and wild-type mice in either the EDL or the SOL muscles (Additional file 6, Figures S5E and S5F). These results demonstrate that *Pparδ*-cKO does not lead to changes in muscle fiber type or oxidative capacity in young animals.

### Old Pparδ-cKO mice become obese and have impaired glucose clearance

**Figure 6:**
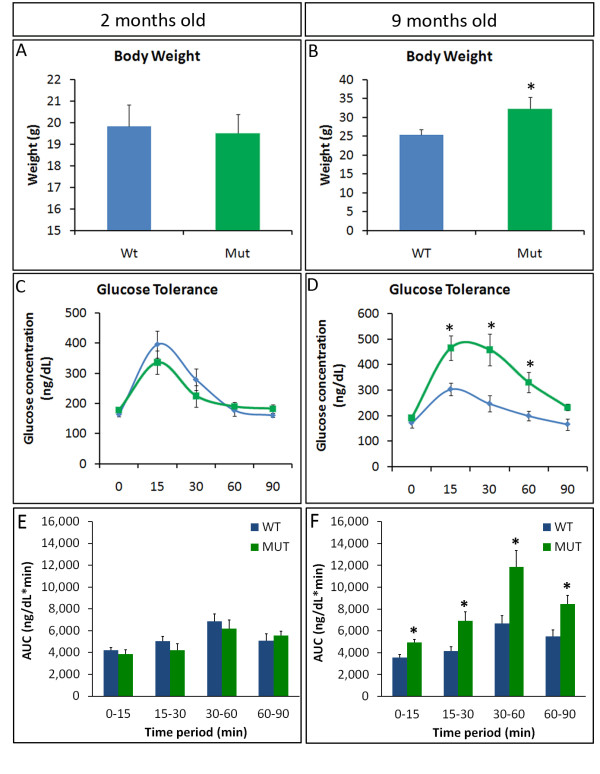
**Age-dependent increases in body weight and glucose tolerance in *Pparδ*-cKO mice**. **(A) **and **(B) **Body weights of female wild-type mice and mutant littermates at two and nine months old (*N *= 6). **(C) **and **(D) **Blood glucose concentration at different time points following intraperitoneal injection of glucose (2 g/kg). The mice were fasted for three hours prior to glucose injection. Glucose concentration was measured using an ACCU-CHEK Active blood glucose meter system (Roche Diagnostics, Indianapolis, IN, USA) (*N *= 3 pairs of female littermates). **(E) **and **(F) **Area under the curve (AUC) analysis the of glucose tolerance test results shown in parts **(C) **and **(D)**, respectively.

PPARδ is an important regulator of muscle energy metabolism. Therefore, we tested the body weight and insulin sensitivity in young and old mutant mice. Young *Pparδ*-cKO mice (two months of age) did not differ significantly in body weight compared to their sex-matched wild-type littermates (Figure 6A). However, older mutant mice (nine months of age) were about 25% heavier on average than their wild-type littermates (Figure 6B). Next we measured the rate of insulin-mediated glucose clearance in the mutant and wild-type mice. The amount of glucose in the blood was measured before (0 minutes) and 15, 30, 60 and 90 minutes after intraperitoneal injection of 2 g/kg glucose (Figures 6C and 6D). Again, young mice showed no differences in the rate of insulin-stimulated glucose uptake as measured by AUC analysis of the glucose tolerance curves (Figure 6E). Strikingly, older mutant mice showed a reduced rate of glucose uptake; their blood glucose levels spiked higher than those of the wild-type mice and took longer to clear from the blood (Figure 6D), resulting in significant increases in the AUC in every time period measured (Figure 6F). These results suggest that the older mice had developed glucose intolerance and an obese phenotype even when fed a normal diet.

## Discussion

The results produced by our *Pparδ*-cKO model establish a previously unappreciated role of PPARδ in skeletal muscle progenitor cell proliferation. The specificity of the *Pparδ*-cKO mouse model is demonstrated by reduced *Pparδ *mRNA expression in both the skeletal muscle and BAT, known to be derived from Myf5 lineage cells. In contrast, *Pparδ *expression was unchanged in WAT, which is derived from a Myf5-independent lineage [20]. The residual *Pparδ *expression detected in *Pparδ*-cKO whole muscle and BAT (10% to 20% of wild-type levels) is probably due to non-Myf5 lineage cells' residing in these tissues, such as nerve cells (neural branches and Schwann cells), vessel-associated cells (blood, lymph, endothelial and smooth muscle cells) and interstitial connective tissue-associated cells (fibroblasts and white adipocytes). In addition, because *Myf5-Cre *is expressed in about 90% of satellite cells [15], a small fraction of *Myf5^- ^*satellite cells would still have normal levels of *Pparδ *expression. We nevertheless observed proliferative defects in satellite cells *in vitro *and muscle regeneration *in vivo*. Further studies using other Cre lines with improved efficiency of *Pparδ *deletion in satellite cells would help to better address the muscle-regenerative defects.

Mice lacking PPARδ in their skeletal muscles are born at normal ratios and are viable. These animals do not appear to have any noticeable defects in body weight or response to glucose early in life. There was no difference in total fiber number or percentage of each fiber type in representative slow (SOL) and fast (EDL) muscles. However, old mice (eight to nine months old) were significantly fatter than their wild-type littermates and were found to have developed glucose intolerance. This phenotype in the aged mouse is similar to that reported in another transgenic mouse model in which conditional mutation of *Pparδ *in mature skeletal muscles is mediated by human α-skeletal actin promoter-driven Cre in the transgenic mouse [33]. In that mouse model (*Pparδ^skm-/-^*), a reduction in the oxidative capacity and muscle fiber-type switching are also detected, whereas such changes are not evident in the *Pparδ*-cKO mouse model used in our study. The discrepancy between these observations may be due to the genetic background and nutrition status (that is, diet composition) of the mice used and the different techniques used for fiber typing. The previous study used qPCR analysis to show increases in *MyHC1 *and *MyHC2b *transcripts in the gastrocnemius muscle [33]. As many muscle fibers express multiple *MyHC *transcripts, small changes in these transcripts may not be sufficient to induce fiber-type switching [46]. In our current study, we used MyHC isoform-specific mAbs to unambiguously identify fiber types, and we examined all fibers within the entire muscle to eliminate bias due to regional clustering of specific fiber types within the same muscle. We have therefore provided strong evidence that *Pparδ*-cKO does not lead to an overt switch of muscle fiber type in young animals based on MyHC isoform protein expression.

We show herein that the expression levels of *Pax7 *are significantly reduced in the whole muscle of *Pparδ*-cKO mice. Because Pax7 is a satellite cell-specific marker [10], this observation suggests a reduction in satellite cell number, which we confirmed by our later results showing that *Pparδ*-cKO mice have fewer satellite cells *in vivo*. Our single-fiber culture model that mimics satellite cell activation *in vivo *further demonstrates that there are fewer proliferating satellite cells and increased numbers of differentiating cells in *Pparδ*-cKO mice. *In vitro *growth and proliferation of *Pparδ*-cKO satellite cell-derived primary myoblasts were also reduced, as shown by the decreased number of cells expressing the cell proliferation marker Ki67 and the increased population doubling time based on the cell growth curve. Conversely, the PPARδ agonist GW501516 stimulates myoblast proliferation. These results provide compelling evidence that PPARδ acts to facilitate satellite cell proliferation.

Recent studies have shown that PPARδ promotes the proliferation of several cell types. PPARδ activation has been shown to increase the proliferation and migration of endothelial progenitors as well as the number of hematopoietic stem cells in the bone marrow [34,47,48]. In addition, PPARδ has been shown to be essential for high glucose-stimulated proliferation of embryonic stem cells [35]. Moreover, PPARδ is an important regulator of the proliferation of several cancer cell types [49,50] (older references are reviewed by Peters and Gonzalez [36]). The PPARδ antagonists SR13904 and GSK0660, which block PPARδ activation, have been shown to reduce cell proliferation [50,51]. The role of PPARδ in cell proliferation has been shown to be due to its activation of the phosphatidylinositol 3-kinase and AKT pathways [34,38], which has been shown to be important in preventing apoptosis and enhancing cell proliferation by triggering cells to overcome cell-cycle arrest and initiating cell proliferation [52,53]. Importantly, AKT signaling plays critical roles in skeletal muscle progenitor cell function and muscle hypotrophy [4,5,54].

A number of other studies have shown that PPARδ promotes differentiation and inhibits (or has no effect on) proliferation in many cell types, particularly keratinocytes, smooth muscle cells, cardiac fibroblasts and several cancer cell lines (extensively reviewed by Peters and Gonzalez [36] and Foreman *et al. *[55]). Therefore, whether PPARδ promotes terminal differentiation or cell proliferation is highly cell type-specific [36]. Intriguingly, investigators in a recent study demonstrated that PPARδ can exert its transcriptional activation or repression both ligand-dependently and ligand-independently in a target-specific manner [56]. Such variations in the mode of PPARδ action probably account for the paradoxical effects of PPARδ in different cell types or even in the same cell type. More research is needed to elucidate how PPARδ regulates satellite cell proliferation and whether it also functions at later stages of myogenesis (see discussion below).

*Pparδ*-cKO animals showed delayed muscle regeneration after injury with CTX. We found that, compared to their wild-type littermates, transgenic mice lacking PPARδ in the skeletal muscle had a reduced proportion of proliferating myoblasts and that the regenerated fibers were smaller in caliber at both five days and two weeks after CTX injury. These results are consistent with our observations that these animals had fewer satellite cells and that *Pparδ*-deficient satellite cells have reduced proliferative potential *in vitro*. Because satellite cells are responsible for skeletal muscle regeneration, reductions in satellite cell number and defective satellite cell proliferation may have resulted in the observed delay in muscle regeneration. In addition to decreased satellite cell proliferation, loss of PPARδ in myofibers may have contributed to the observed defects in muscle regeneration. Moreover, other signaling mechanisms may have compensated for the loss of PPARδ in our conditional mutant, leading to completion of muscle regeneration at a later time. Coincidentally, the incidence of sarcopenia (age-related muscle loss) has been shown to correlate with reduced levels of PPARδ, and pharmacological activation of PPARδ has been shown to reduce the incidence of sarcopenia by increasing nuclear accretion in myofibers [57]. These results support the notion that PPARδ is important for proper skeletal muscle regeneration in response to injury or daily wear and tear (maintenance) by increasing satellite cell proliferation and promoting fusion of differentiated myocytes *in vivo*.

We examined the expression levels of several previously identified PPARδ target genes to understand how PPAR functions in skeletal muscle and satellite cells. Our results show that the expression of both *mCPT1β *and *FoxO1 *is reduced in mature uninjured muscles of the *Pparδ*-cKO mice, suggesting that they are molecular targets of PPARδ under resting conditions. Since PPARδ has been shown by many groups to be a regulator of oxidative capacity in skeletal muscle [21], it is not surprising that key players in the lipid oxidation pathway would be downregulated after removal of PPARδ. Interestingly, *mCPT1β *expression is not significantly reduced in *Pparδ*-cKO muscle at day 5 during regeneration. At this time point, degenerated muscle fibers have not been replaced by new fibers and myoblast proliferation is at the peak stage [8]. This result seems to suggest that *mCPT1β *is a target of PPARδ in mature resting muscles, but not in proliferating myoblasts. Of all the PPARδ target genes we examined, *FoxO1 *was the only gene whose expression was downregulated in both resting and regenerating muscles of *Pparδ*-cKO mice. The downregulation of *FoxO1 *in *Pparδ*-cKO regenerating muscle indicates that FoxO1 may act at downstream of PPARδ in regulating myoblast proliferation and inhibiting its differentiation. Indeed, it has been shown that constitutive activation of FoxO1 inhibits myoblast differentiation [44]. However, FoxO1 also acts at later stages of myogenesis, during the fusion of differentiated myocytes into myotubes [58]. These previous studies and our present results suggest that PPARδ acts through FoxO1 in both satellite cells and mature muscle fibers but has distinct functions in these cell types. It regulates the proliferation of satellite cells and fusion of muscle fibers. These combined effects of PPARδ's acting through FoxO1 explain the regenerative defects seen in the *Pparδ*-cKO muscles.

PPARδ has also been implicated in the treatment of degenerative muscle diseases such as Duchenne muscular dystrophy (DMD), which is caused by the mutation of the *dystrophin *gene [59,60]. One research group showed that PPARδ activation by the agonist GW501516 increased the expression of utrophin A, a key member of the dystrophin-associated protein complex, in the C_2_C_12 _myoblast cell line [61]. PPARδ has been suggested to be a direct transcriptional regulator of *utrophin A in vivo *[43]. Intriguingly, utrophin A overexpression can improve the integrity of the sarcolemma, protect muscles from contraction induced damage and help to alleviate muscle wasting and slow down the disease progression of DMD [62]. Therefore, understanding how PPARδ functions in skeletal muscle tissue and its progenitor cells has important implications for muscle regeneration and the treatment of degenerative muscle diseases.

## Conclusions

Our *in vivo *and *in vitro *analyses of myogenic lineage specific *Pparδ *gene knockouts demonstrate a critical role of PPARδ in satellite cell proliferation and postnatal regeneration of skeletal muscles. In addition, we provide evidence for a role of PPARδ in regulating muscle insulin resistance, as indicated by the glucose tolerance test results. However, we were unable to detect overt changes in skeletal muscle fiber types by MyHC isoform-specific antibody labeling. These results not only support the previously established role of PPARδ in muscle energy metabolism and insulin sensitivity but also demonstrate a novel role of PPARδ in muscle progenitor cell function. These results have implications for the treatment of muscular dystrophies and muscle-wasting conditions by targeting PPARδ signaling at the stem cell level.

## Abbreviations

BSA: bovine serum albumin; DMEM: Dulbecco's modified Eagle's medium; H & E: hematoxylin and eosin; mAb: monoclonal antibody; PBS: phosphate-buffered saline; PCR: polymerase chain reaction.

## Competing interests

The authors declare that they have no competing interests.

## Authors' contributions

AA carried out the mouse and cell culture studies, analyzed the data and drafted the manuscript. CJ carried out part of the muscle regeneration and immunofluorescence labeling studies. DP conducted the Western blot analysis. YW provided the *Pparδ*-floxedmice, analyzed the data and revised the manuscript. SK conceived, designed and coordinated the study; analyzed the data; and revised the manuscript. All authors read and approved the final manuscript.

## Supplementary Material

Additional file 1 **Table S1 Primer sequences for quantitative PCR**. Click here for file

Additional file 2 **Figure S1 *Pparδ*-cKO strategy**. **(A) **Peroxisome proliferator-activated receptor δ (*Pparδ*) gene structure with exons numbered sequentially. Note that LoxP sequences are inserted before and after exon 4 encoding the DNA-binding domain of PPARδ. In the presence of Cre (driven by Myf5 locus in this study), exon 4 is excised, resulting in premature stop in translation and generation of a short, truncated protein without a DNA-binding domain. **(B) **Representative Western blot showing the relative expression of PPARδ protein in the wild-type (WT) and *Pparδ*-conditional knockout (*Pparδ*-cKO) gastrocnemius muscles. The upper nonspecific band serves as an indicator of the relative amount of total protein loaded onto the gel. Click here for file

Additional file 3 **Figure S2 Relative expression of PPARδ target genes in mature noninjured muscles**. RNA samples isolated from the tibialis anterior (TA) muscles of six-week old mice were used for quantitative PCR analysis. **(A) ***UCP1*. **(B) ***Sirt1*. **(C) ***PGC1α. N *= 4 for *UCP1 *and *Sirt1 *and *N *= 6 for *PGC1α*. Click here for file

Additional file 4 **Figure S3 Satellite cell (A) and myonuclei (B) abundance in extensor digitorum longus fibers of wild-type and *Pparδ*-cKO mice during postnatal growth at three and five weeks old**. Two pairs of mice at each age were used. The satellite cell number was averaged from 30 to 37 fibers, and myonuclei were averaged from 20 to 40 fibers. Click here for file

Additional file 5 **Figure S4 Role of *Pparδ *in primary myoblast proliferation**. **(A) **Relative expression of the peroxisome proliferator-activated receptor δ (*Pparδ*) gene in mutant and wild-type myoblasts at passages 3 and 4 (*N *= 4). **(B) **Percentage of proliferating cells (Ki67^+^) in wild-type primary myoblasts at 24 hours after control (dimethyl sulfoxide vehicle) and 100 nM PPARδ agonist GW501516 treatments (*N *= 10). Click here for file

Additional file 6 **Figure S5 NADH-tetrazolium reductase staining showing the relative abundance of oxidative fibers in *Pparδ*-cKO and wild-type skeletal muscles**. Staining shows the three different fiber types. Oxidative fibers (type 1) are darkly stained, intermediate fibers (type 2a) are moderately stained and glycolytic fibers (types 2b and 2x) are unstained. **(A) **and **(B) **Representative images of fast muscles (extensor digitorum longus (EDL)). **(C) **and **(D) **Representative images of slow muscles (soleus (SOL)). **(E) **and **(F) **Relative nicotinamide adenine dinucleotide, reduced (NADH), intensity levels between peroxisome proliferator-activated receptor δ (PPARδ) wild-type and PPARδ-conditional knockout (*Pparδ*-cKO) animals at six weeks of age (*N *= 3). Click here for file
