# Supplementary material for: PPARδ regulates satellite cell proliferation and skeletal muscle regeneration
Source: Skelet Muscle. 2011 Nov 1;1:33. doi: 10.1186/2044-5040-1-33 (PMC3223495; doi:10.1186/2044-5040-1-33)
Supplement: Additional file 1 — Table S1 Primer sequences for quantitative PCR. [file 2044-5040-1-33-S1.DOCX]

Supplementary Table 1: Primer sequences for qPCR

| qPCR primers | forward primer (5'-3') | reverse Primer (5'-3') |
| --- | --- | --- |
| Pparδ | GACCAGAACACACGCTTCCT | CCGACATTCCATGTTGAGG |
| Pparα | ACAAGGCCTCAGGGTACCA | GCCGAAAGAAGCCCTTACAG |
| Pparγ | CAAGAATACCAAAGTGCGATC | GAGCTGGGTCTTTTCAGAATAATAAG |
| Pax7 | GCTACCAGTACAGCCAGTATG | GTCACTAAGCATGGGTAGATG |
| MYH | AGTCCCAGGTCAACAAGCTG | Common F primer, individual R primers |
| MYH7 (MyHC1) | Common F primer | TCCCACCTAAAGGGCTGTTG |
| MYH2 (MyHC2a) | Common F primer | GCATGACCAAAGGTTTCACA |
| MYH4 (MyHC2b) | Common F primer | TTTCTCCTGTCACCTCTCAACA |
| MYH1 (MyHC2x) | Common F primer | CACATTTGGCTCATCTCTTGG |
| UCP1 | GTGAAGGTCAGAATGCAAGC | AGGGCCCCCTTCATGAGGTC |
| Pgc1α | AACCACACCCACAGGATCAGA | TCTTCGCTTTATTGCTCCATGA |
| FoxO1 | CTGGGTGTCAGGCTAAGAGT | GGGGTGAAGGGCATCTTT |
| mCPT1β | GGGCACCTCTGGGAGTTTGT | TGGCTCACCCACACAGTGT |
| Sirt1 | AGCAGGTTGCAGGAATCCAA | CACGAACAGCTTCACAATCAACTT |
