# Supplementary figures and images for: PPARδ regulates satellite cell proliferation and skeletal muscle regeneration
Source: Skelet Muscle. 2011 Nov 1;1:33. doi: 10.1186/2044-5040-1-33 (PMC3223495; doi:10.1186/2044-5040-1-33)

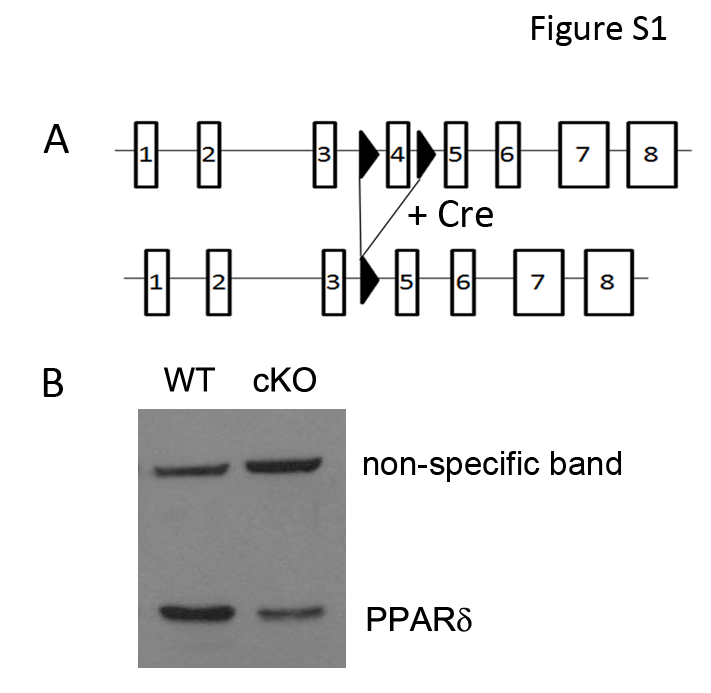

Supplement: Additional file 2 — Figure S1 Pparδ-cKO strategy. (A) Peroxisome proliferator-activated receptor δ (Pparδ) gene structure with exons numbered sequentially. Note that LoxP sequences are inserted before and after exon 4 encoding the DNA-binding domain of PPARδ. In the presence of Cre (driven by Myf5 locus in this study), exon 4 is excised, resulting in premature stop in translation and generation of a short, truncated protein without a DNA-binding domain. (B) Representative Western blot showing the relative expression of PPARδ protein in the wild-type (WT) and Pparδ-conditional knockout (Pparδ-cKO) gastrocnemius muscles. The upper nonspecific band serves as an indicator of the relative amount of total protein loaded onto the gel. [file 2044-5040-1-33-S2.TIFF]

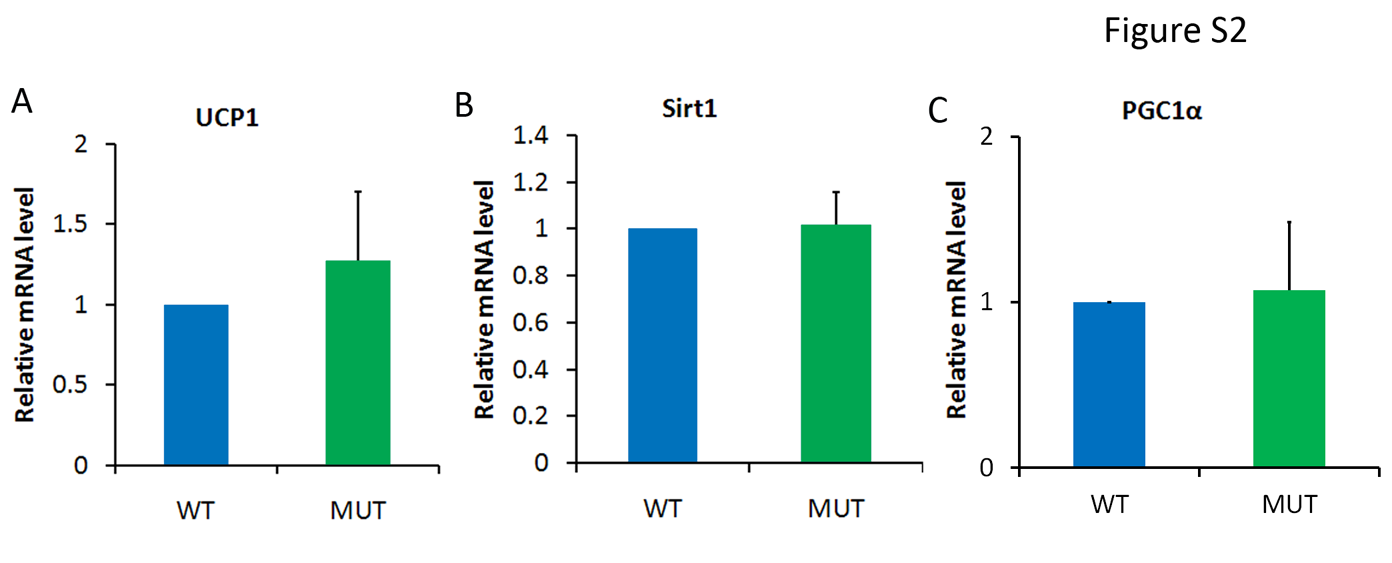

Supplement: Additional file 3 — Figure S2 Relative expression of PPARδ target genes in mature noninjured muscles. RNA samples isolated from the tibialis anterior (TA) muscles of six-week old mice were used for quantitative PCR analysis. (A) UCP1. (B) Sirt1. (C) PGC1α. N = 4 for UCP1 and Sirt1 and N = 6 for PGC1α. [file 2044-5040-1-33-S3.TIFF]

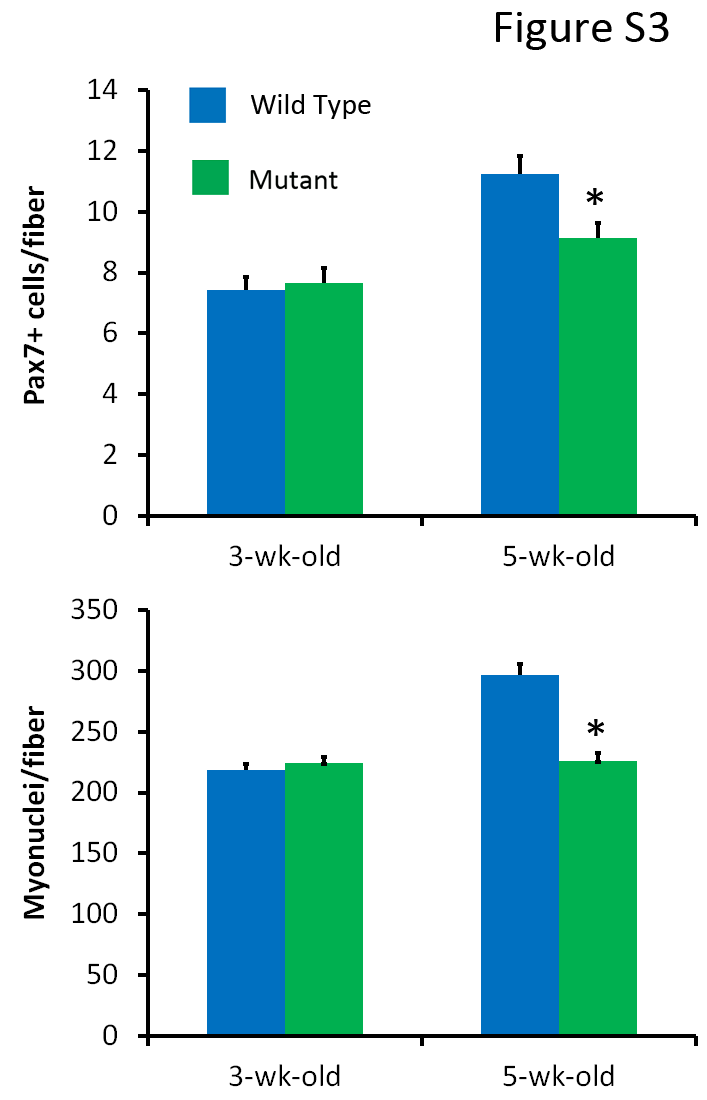

Supplement: Additional file 4 — Figure S3 Satellite cell (A) and myonuclei (B) abundance in extensor digitorum longus fibers of wild-type and Pparδ-cKO mice during postnatal growth at three and five weeks old. Two pairs of mice at each age were used. The satellite cell number was averaged from 30 to 37 fibers, and myonuclei were averaged from 20 to 40 fibers. [file 2044-5040-1-33-S4.TIFF]

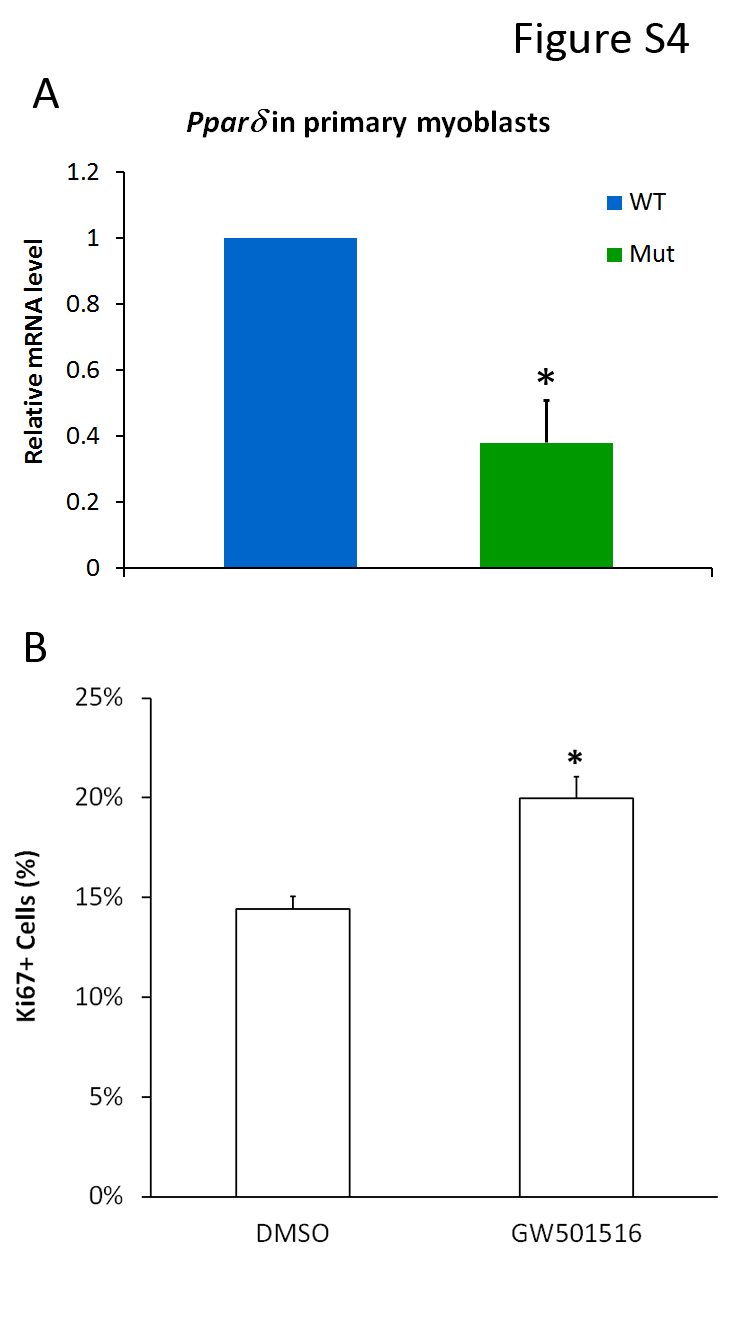

Supplement: Additional file 5 — Figure S4 Role of Pparδ in primary myoblast proliferation. (A) Relative expression of the peroxisome proliferator-activated receptor δ (Pparδ) gene in mutant and wild-type myoblasts at passages 3 and 4 (N = 4). (B) Percentage of proliferating cells (Ki67+) in wild-type primary myoblasts at 24 hours after control (dimethyl sulfoxide vehicle) and 100 nM PPARδ agonist GW501516 treatments (N = 10). [file 2044-5040-1-33-S5.TIFF]

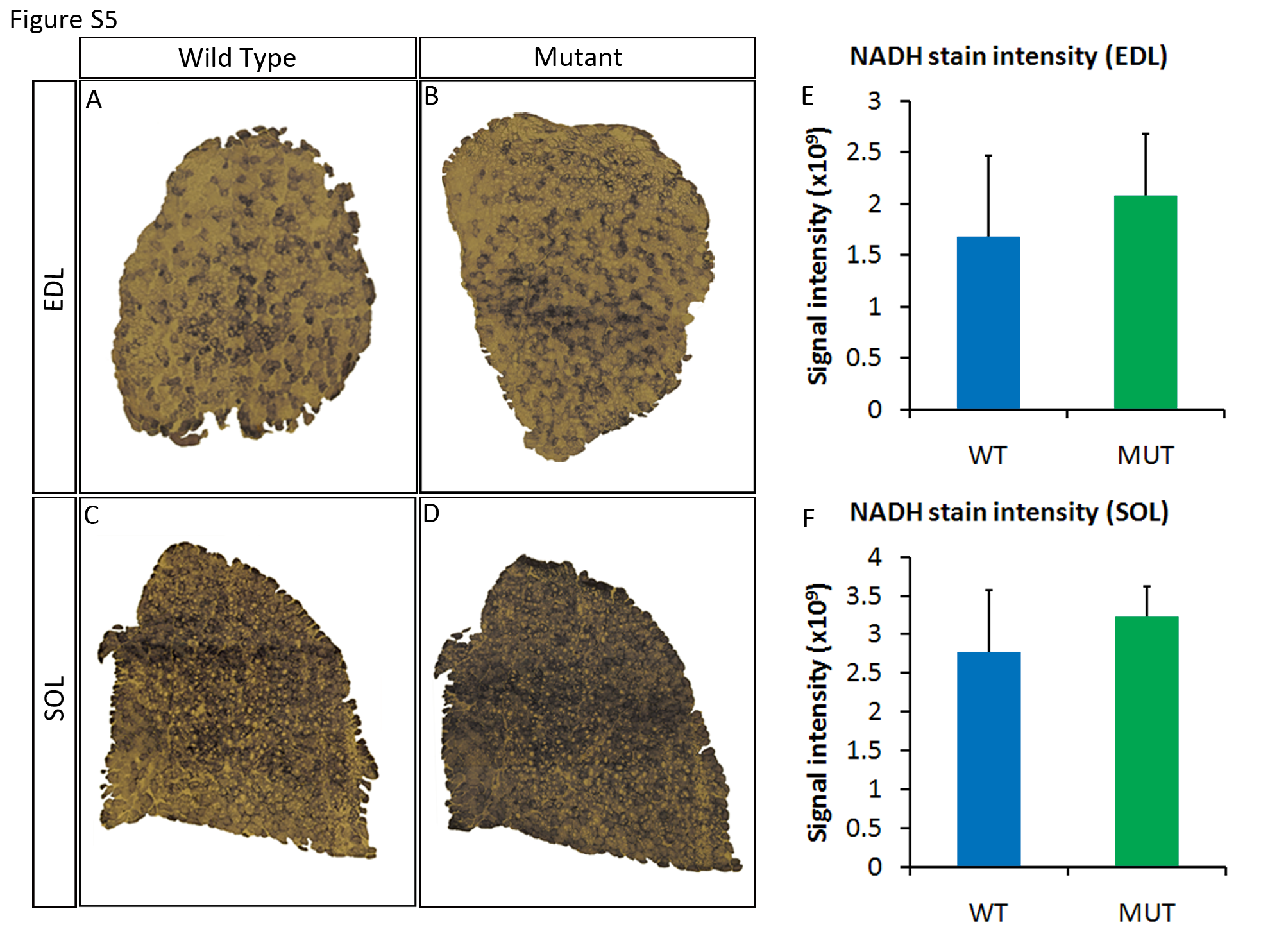

Supplement: Additional file 6 — Figure S5 NADH-tetrazolium reductase staining showing the relative abundance of oxidative fibers in Pparδ-cKO and wild-type skeletal muscles. Staining shows the three different fiber types. Oxidative fibers (type 1) are darkly stained, intermediate fibers (type 2a) are moderately stained and glycolytic fibers (types 2b and 2x) are unstained. (A) and (B) Representative images of fast muscles (extensor digitorum longus (EDL)). (C) and (D) Representative images of slow muscles (soleus (SOL)). (E) and (F) Relative nicotinamide adenine dinucleotide, reduced (NADH), intensity levels between peroxisome proliferator-activated receptor δ (PPARδ) wild-type and PPARδ-conditional knockout (Pparδ-cKO) animals at six weeks of age (N = 3). [file 2044-5040-1-33-S6.TIFF]
